# Stability of Carbon Supported Silver Electrocatalysts for Alkaline Oxygen Reduction and Evolution Reactions

**DOI:** 10.1021/acsaem.3c01717

**Published:** 2023-11-10

**Authors:** Jonas Mart Linge, Valentín Briega-Martos, Andreas Hutzler, Birk Fritsch, Heiki Erikson, Kaido Tammeveski, Serhiy Cherevko

**Affiliations:** † Institute of Chemistry, University of Tartu, Ravila 14a, 50411 Tartu, Estonia; ‡ Helmholtz Institute Erlangen-Nürnberg for Renewable Energy (IEK-11), Forschungszentrum Jülich GmbH, Cauerstrasse 1, 91058 Erlangen, Germany

**Keywords:** Stability, Electrocatalysis, Fuel cells, IL-TEM, Mass spectrometry, Silver catalyst, Carbon support, Oxygen reduction

## Abstract

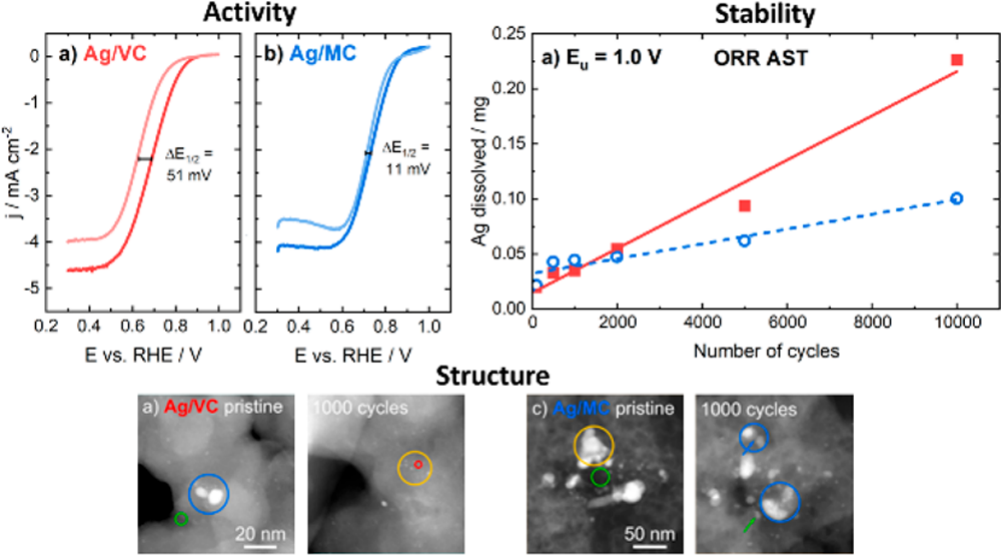

Ag-based electrocatalysts are promising candidates to catalyze the sluggish oxygen reduction reaction (ORR) in anion exchange membrane fuel cells (AEMFC) and oxygen evolution reaction (OER) in unitized regenerative fuel cells. However, to be competitive with existing technologies, the AEMFC with Ag electrocatalyst must demonstrate superior performance and long-term durability. The latter implies that the catalyst must be stable, withstanding harsh oxidizing conditions. Moreover, since Ag is typically supported by carbon, the strict stability requirements extend to the whole Ag/C catalyst. In this work, Ag supported on Vulcan carbon (Ag/VC) and mesoporous carbon (Ag/MC) materials is synthesized, and their electrochemical stability is studied using a family of complementary techniques. We first employ an online scanning flow cell combined with inductively coupled plasma mass spectrometry (SFC-ICP-MS) to estimate the kinetic dissolution stability window of Ag. Strong correlations between voltammetric features and the dissolution processes are discovered. Very high silver dissolution during the OER renders this material impractical for regenerative fuel cell applications. To address Ag stability during AEMFC load cycles, accelerated stress tests (ASTs) in O_2_-saturated solutions are carried out in rotating disk electrode (RDE) and rotating ring-disk electrode (RRDE) setups. Besides tracking the ORR performance evolution, an *ex situ* long-term Ag dissolution study is performed. Moreover, morphological changes in the catalyst/support are tracked by identical-location transmission electron microscopy (RDE-IL-TEM). Voltammetry analysis before and after AST reveals a smaller change in ORR activity for Ag/MC, confirming its higher stability. RRDE results reveal a higher increase in the H_2_O_2_ yield for Ag/VC after the ASTs. The RDE-IL-TEM measurements demonstrate different degradation processes that can explain the changes in the long term performance. The results in this work point out that the stability of carbon-supported Ag catalysts depends strongly on the morphology of the Ag nanoparticles, which, in turn, can be tuned depending on the chosen carbon support and synthesis method.

## 1 Introduction

There is a push for greener energy worldwide to abandon fossil fuels by 2050. The primary alternative energy sources are the sun and wind.^1,2^ However, these are intermittent energy sources. This behavior prompts us to save the surplus energy for later use. One way is to turn this excess into hydrogen and oxygen by water electrolysis and transport and use hydrogen on demand at any time. Hydrogen can be transformed to energy through proton exchange membrane fuel cells (PEMFC), anion exchange membrane fuel cells (AEMFC), and 2-in-1 reversible fuel cells (also known as unitized regenerative fuel cells, URFC).^3−5^ The latter technology allows hydrogen production through electrolysis and direct consumption via PEMFC or AEMFC.

The URFC device is more compact than a combination of electrolysis and fuel cell systems separately, costs less and has higher specific energy density.^6,7^ However, it still lacks catalysts for oxygen reduction and evolution reactions (ORR and OER) that are more efficient, durable and at a lower cost.^8^ On the one hand, Pt is commonly employed as the catalyst on the cathode since it is the best pure metal for the ORR.^9^ On the other hand, more active and stable Ir oxide catalysts constitute the anode for the OER.^2,10−12^ Ir oxide and Pt also serve as hydrogen oxidation and hydrogen evolution reaction catalysts when the URFC operates as a fuel cell and electrolyzer, respectively. These rare and expensive state-of-the-art noble metal catalysts make the URFC technology expensive.^1,10,13^

Another significant limitation is the stability of these catalysts.^14,15^ The electrocatalytic materials undergo primary and secondary degradation mechanisms. The primary degradation mechanisms comprise dissolution of the active material itself and corrosion of the carbon support. The main secondary degradation mechanisms are coalescence, agglomeration, and detachment of catalyst particles, as well as Ostwald ripening.^16^ For investigating primary degradation mechanisms, methods such as online electrochemical inductively coupled plasma mass spectrometry (ICP-MS) for the quantification of dissolved species in the form of ions in the solution and online electrochemical mass spectrometry for the detection of volatile products (OLEMS) can be employed.^17,18^ In contrast, secondary degradation mechanisms can be inspected by using identical-location transmission electron microscopy (IL-TEM).^19^

Due to the high prices of Pt and Ir, many other potential catalysts that are more favorable, comparably active, and stable for both the ORR and the OER are still being searched. Ag is postulated as a possible alternative, since it is less expensive and shows a comparable ORR electrocatalytic activity to Pt in alkaline conditions.^20,21^ Ag has been used in Siemens fuel cells in combination with nickel and titanium, and voltages around 800 and 900 mV at current densities of 400 and 200 mA cm^–2^ have been achieved, respectively.^22^ With gas diffusion electrodes (GDEs) made of Raney-silver and PTFE (polytetrafluoroethylene), voltages above 1 V and current densities around 30 mA cm^–2^ have been reached.^23^

Although silver is a good catalyst in fuel cells, during electrolysis it can present more difficulties since it tends to be permanently oxidized and eventually dissolve at the required higher potentials.^23^ Nevertheless, it can perform with a good round-trip efficiency in bifunctional alkaline fuel cells, which is a property of rechargeable Li-ion batteries.^23^ For application in URFCs, Ag has been combined with Ti and it was found that Ti–Ag/Ti (Ti–Ag film coated on Ti) showed good corrosion resistance that is comparable to bare Ti at high potentials and retained its current densities during long working mode at 2.0 V vs NHE under constant air purging.^24^

Besides URFCs, Ag based catalysts have displayed different activities in AEMFCs. For example, when Ag was deposited onto activated carbon (10 wt % Ag), the resulting catalyst achieved peak power density of 109 mW cm^–2^ and a 60 wt % Ag/CB catalyst showed 330 mW cm^–2^ and 506 mW cm^–2^ in a more recent work.^25−27^ To withstand the higher upper potential limits required in the URFCs for the successful electrolysis of water during the electrolysis mode and later for the ORR during the fuel cell mode, the high stability of the catalyst is of utmost importance for both modes.

Apart from activity, stability is another important parameter when establishing the suitability of an electrocatalyst for its application in electrochemical energy conversion devices. Ag stability depends on electrochemical oxidation processes occurring at the surface of the catalysts. Understanding them is crucial for developing new, more stable electrocatalysts. For example, it is affected by electrochemical oxidation in the anodic direction, during which different Ag oxides are formed depending on the employed upper limit potential. The thermodynamic data for Ag oxidation processes in standard conditions is well predicted by the respective Pourbaix diagram^28^ as follows (the indicated potentials are SHE-scaled at pH 13):
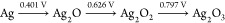
However, electrochemical data point out that the above-mentioned processes can explain only some of the observed anodic peak in the commonly obtained cyclic voltammograms. The formation of dissolved species such as AgO^–^ or Ag(OH)_2_^–^ must also to be considered.^29^ Furthermore, during the negative-going potential sweep the Ag surface (partially) depassivates by the reduction of the surfaces oxides, and during this processes cathodic Ag dissolution also takes place as shown by online electrochemical ICP-MS measurements by Schalenbach et al.^17^

Ag has exhibited good results in previous stability tests in 0.1 M KOH. For example, in a simulated stability test where potential was cycled in the ORR potential region for 30,000 s of continuous operation, Ag lost only 7.1% of its current density compared to 16% for Pt/C.^30^ Ag-PBMO_5_ (Ag–Pr_0.95_Ba_0.95_Mn2O_5-δ_, engineered perovskite nanofibers) stability was tested by performing 10,000 potential LSV cycles at an electrode rotation speed of 1600 rpm and its half-wave potential (*E*_1/2_) shifted by 23 mV (42 mV for Pt/C). In the same study, the chronoamperometric tests for 50,000 s show that the Ag catalyst only lost around 9% of its relative current density, while the Pt/C lost 72% under same testing conditions.^31^ Another study reported a chronoamperometric test for 25,000 s after which the Ag catalyst retained 80.3% of its initial current compared to 39.2% of Pt/C.^32^ When the potential was cycled for 1,000 times in a potential range from −1.3 to 0 V vs SCE in O_2_-saturated 0.1 M KOH solution, *E*_1/2_ shifted only by 36 mV for the tested Ag catalyst.^33^ Also, Ag catalysts have been investigated for stability in actual fuel cells. For example, an AEMFC was run at 250 mA cm^–2^ in H_2_/air (CO_2_-free) environment, and after 10 h the voltage decreased by 15%.^27^ Nevertheless, in all the works described above, the degradation mechanisms are only speculated about and only a few specific experiments have been carried out to directly determine the dissolution of Ag in the catalyst materials.^17^

Taking all above into consideration, this work focuses on investigating the dissolution contribution on the degradation behavior of Ag-based electrocatalysts toward the oxygen reduction and evolution reactions. One of the employed method is the coupling of the electrochemical scanning flow cell to the ICP-MS (SFC-ICP-MS). This experimental setup provides time and potential-resolved dissolution information.^18^ Although this technique cannot determine the oxidation state of the dissolved elements,^34^ it is extremely helpful for the determination of dissolution mechanisms due to the potential-resolved dissolution data.

However, it is not possible to perform long-term experiments for studying the stability of the catalyst near operating conditions by means of SFC-ICP-MS. In this regard, the rotating disk electrode (RDE) method can be used as it allows to perform longer experiments than SFC-ICP-MS, and bulk samples can be taken during accelerated stress tests (ASTs), while providing better mass transfer on the electrode.^35^

In this work, both SFC (online) and RDE (offline) methods are chosen in combination with ICP-MS to study the stability of Ag electrocatalysts. The Ag-based materials are prepared on mesoporous carbon (MC) support that possesses well-defined and uniform bimodal mesopores with pore sizes of 7 and 25 nm (ECS004201, Pajarito Powder, LLC) and on conventional Vulcan carbon (VC).^36^ Rotating ring-disk electrode (RRDE) measurements were also carried out to obtain further insights into the reasons behind the activity decrease after the ASTs.

Transmission electron microscopy was employed to analyze the initial morphologies of the electrocatalyst as well as their evolution during the ASTs by using the RDE identical-location approach (RDE-IL-TEM). The Ag/MC catalyst showed better stability at different electrochemical conditions than Ag/VC. The results point out that the applicability of carbon-supported Ag electrocatalysts in URFC is dramatically compromised due to the remarkably high dissolution at the working potentials for the OER. The TEM imaging suggests that the synthetic route of the electrocatalyst and the resulting morphology play a critical role on the activity and the dissolution behavior during the operating conditions.

## 2 Experimental Section

### 2.1 Materials Preparation

The Ag nanoparticles were prepared by one-pot wet chemical synthesis using hydrazine hydrate (H_4_N_2_·H_2_O, 64%, Arcos Organics) as the reducing agent in the presence of nitrogen-doped bimodal mesoporous carbon support (ECS004201, Pajarito Powder, LLC). Vulcan carbon XC-72R (Cabot Corp.) supported Ag catalyst was used as a reference material. In brief, the carbon support material was suspended in Milli-Q water, then citrate tribasic dihydrate (Fluka, puriss p.a. ≥ 99%) and silver precursor (AgNO_3_, ≥ 99%, Sigma-Aldrich) were added, and the synthesis solution was stirred on a magnetic stirrer for 0.5 h. Then suspension was heated to 50 °C and reducing agent was added (hydrazine hydrate). The synthesis mixture was further stirred until yellow color disappeared. The resulting catalysts with a nominal 40 wt % Ag loading were washed, vacuum-filtered, and left overnight in an oven at 60 °C to dry. The synthesis is more thoroughly described elsewhere,^37,38^ and additional physical characterization can be found in the previous works.^36,38^

### 2.2 IL-TEM Characterization

IL-TEM characterization was carried out using a Talos F200i instrument (Thermo Fisher Scientific), which was operated at a primary electron energy of 200 keV in scanning TEM (STEM) mode. A high-angle annular dark field (HAADF) detector was utilized to exploit the mass–thickness contrast between the Ag electrocatalyst and its VC/MC support material. Spectrum images using energy-dispersive X-ray spectroscopy (STEM-EDXS) were recorded with a Dual Bruker XFlash 6|100 EDS detector.

### 2.3 Online Electrochemical Dissolution Measurements

An inductively coupled plasma mass spectrometer (ICP-MS, PerkinElmer, Nexion 350X) together with a three-electrode scanning flow cell (SFC) were used for the online electrochemical dissolution measurements employing a custom-made LabVIEW software.^39^ The potentiostat was a Gamry Reference 600. The working electrode was a 5 × 5 cm glassy carbon (GC) plate covered with catalyst spots with a radius ranging from 600 to 750 μm, which were prepared by drop casting 0.2 μL of catalyst ink with concentration of 1 mg mL^–1^. Before each measurement, the GC plate was polished with an alumina slurry with a grain size of 0.3 μm. The counter electrode was a GC rod with a diameter of 5 mm (HTW Sigradur G). The reference electrode was Ag/AgCl/3 M KCl electrode (Metrohm), and it is situated in the outlet channel of the SFC system to avoid contamination with chloride ions. The potential values reported in this paper are given with respect to the reversible hydrogen electrode (RHE). The experiments were carried out in an alkaline environment where the electrolyte was 0.05 M KOH solution prepared from KOH pellets (Sigma-Aldrich, p.a.) with a pH of approximately 12.7. The electrolyte solution was purged with Ar gas. The average flow rate from SFC to ICP-MS is 3.5 μL s^–1^. The time delay of electrolyte flow from the SFC to ICP-MS was accounted for and adjusted for the direct correlation between the potential and dissolution data. Before the measurements, the ICP-MS was calibrated using 0.5, 1, and 5 ppb solutions of a Ag standard. Before the electrolyte entered the ICP-MS it was mixed 1:1 with a 1% HNO_3_ solution with 10 ppb internal standard (Rh) solution.

Two different electrochemical protocols were chosen for these measurements. In *Protocol 1*, consecutive cyclic voltammograms were performed at a scan rate of 10 mV s^–1^, with a lower potential limit of 0.3 V vs RHE in all cases and an upper potential limit that was increased each cycle by 0.1 V going from 0.9 to 1.8 V vs RHE. In *Protocol 2*, two consecutive cyclic voltammograms at a scan rate of 2 mV s^–1^ were performed, both with a lower potential limit of 0.3 V vs RHE and an upper potential limit of 1.8 V.

### 2.4 Activity and Ex Situ Dissolution Measurements Using RDE Configuration

For RDE measurements, a three-electrode system was employed in a PTFE electrochemical cell, especially designed for long-term dissolution experiments. The working electrode was a glassy carbon (GC) disk embedded in a PTFE cylinder (AFE5T050GC, Pine Research) and its geometric surface area was ca. 0.196 cm^2^, onto which catalyst ink was drop casted to have catalyst loading of 150 μg cm^–2^. The counter electrode was a GC rod. The reference electrode was a Ag/AgCl/3 M KCl electrode (Metrohm), analogously to the SFC-ICP-MS measurements. The ORR experiments were carried out in O_2_-saturated 0.05 M KOH solution to use the same conditions as for SFC-ICP-MS. The electrode rotation rate (ω) was set constant at 960 rpm with a MSR rotator (Pine Research) for the whole measurement. During the AST of 10,000 potential cycles in the potential window of 0.3 and 1.0 V (or 1.2 V) vs RHE, 2 mL samples of the working electrolyte were collected at the 100th cycle, 500th cycle, 1,000th cycle, 2,000th cycle, 5,000th cycle, and 10,000th cycle, which were later measured with the ICP-MS for determining the Ag concentration in solution. The working electrolyte volume was restored to the original value with fresh electrolyte after taking each sample, and this was considered in the calculations for the Ag concentration. The ORR polarization curves were recorded before and after AST at 10 mV s^–1^ to evaluate the catalyst activity. A Gamry Reference 600 potentiostat was used for these measurements.

### 2.5 RRDE Measurements

Rotating ring disk electrode (RRDE) measurements were carried out using a two-channel SP-300 potentiostat (BioLogic). One channel was used for the GC disk electrode, and the other was connected to the Pt ring (AFE79R9GCPT, Pine Research). A MSR rotator (Pine Research) was used for these measurements. The potential of the Pt ring was maintained at 1.1 V vs RHE, since H_2_O_2_ oxidation is controlled by mass-transport at this value, allowing its quantitative detection.

### 2.6 RDE-IL-TEM Measurements

A custom-made RDE tip based on previous works was employed for RDE-IL-TEM experiments.^40−42^ A 5 mm GC disk embedded in a custom-made PEEK holder was used as the working electrode. One μL of the Ag catalyst ink used for the RDE experiments diluted 10-fold were drop casted on an Au TEM grid (Maxtaform finder grid) of 3.05 mm diameter. This TEM grid was centered on top of the GC disk and fixed by using a PEEK cap with a 2.95 mm centered opening, which was screwed to the PEEK holder. In this way, the Au TEM grid with the deposited Ag catalyst was fixed and in contact to the GC disk, making it possible to perform the electrochemical ASTs in an analogous way as described in the previous section. The onset of Au dissolution is higher than 1.2 V vs RHE in alkaline media. Since the RDE-IL-TEM measurements were performed only up to 1.0 V vs RHE, Au dissolution will not take place and any possible Au contamination can be discarded.^43^ An EDI101 rotator and CTV101 speed control unit (Radiometer Analytical) were used for these measurements.

HAADF-STEM images of the Au grid with the deposited Ag catalyst were acquired prior to the electrochemical measurements using markers on the TEM grid to locate regions of interest in the sample. After that, the TEM grid was placed in the RDE and 500 cycles of the AST were carried out. The TEM grid was then removed, and images were taken at the same locations used before the electrochemical AST. This procedure was repeated after 500 more cycles of AST, making a total of 1,000 cycles for the third set of micrographs.

### 2.7 Calculation of the Pourbaix Diagrams

The Pourbaix diagrams shown in this work have been calculated and built using the data found in ref (28). The horizontal and tilted lines of the different electrochemical processes were drawn from the corresponding Nernst equations. While the horizontal lines come from processes for which the concentration of protons (or hydroxides) does not appear in the Nernst equation, the reactions with tilted lines are pH dependent. Special attention was paid to the pH values at which lines for different reactions cross each other, which were also used for plotting the diagrams. Two different Pourbaix diagrams were built using 10^–4^ and 10^–9^ M, respectively, as concentration values for all soluble species in each case. These values were introduced into the Nernst equations when necessary. In the case of chemical processes without the transfer of electrons, the vertical lines in the Pourbaix diagram are determined by the equilibrium constant of the given process.

## 3 Results and Discussion

### 3.1 STEM Characterization

**Figure 1:**
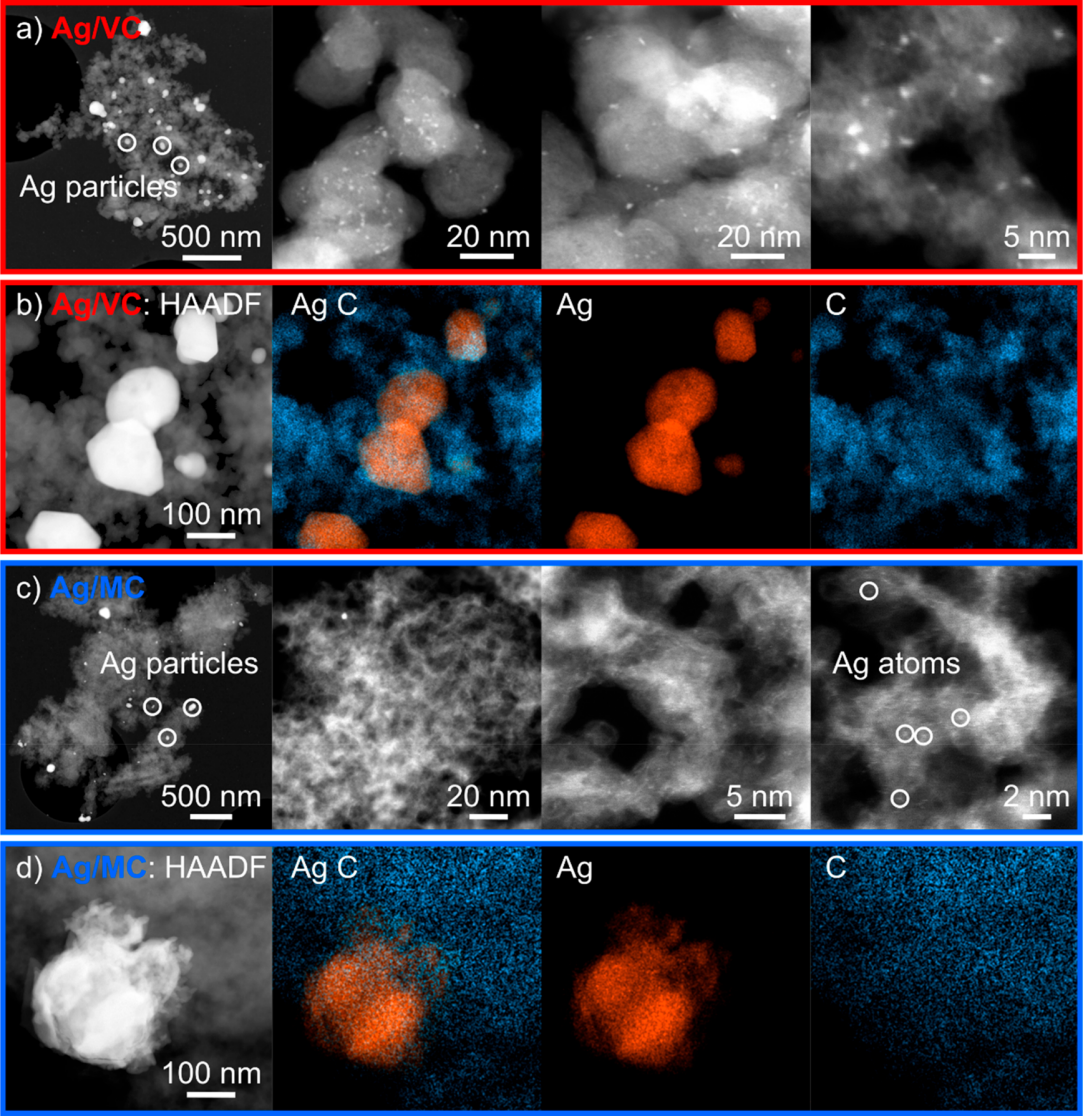
Comparison between Ag/VC (a, b) and Ag/MC (c, d), with HAADF-STEM (a, c) and STEM-EDX spectrum images (b, d), showcasing the elemental distribution of Ag (orange) and C (blue). Note that for MC, high-resolution STEM revealed single atomic clusters (marked with circles).

Comprehensive materials characterizations of the samples studied here, Ag/VC and Ag/MC, can be found in the previous work by Linge et al., under the designation of Ag/VC_HH and Ag/4201_HH, respectively.^38^ In the present work, further characterization by HAADF-STEM and spectrum imaging is additionally performed (Figure 1).

At low magnification (Figures 1a and 1c with 500 nm scale), the size and morphology of the Ag particles appear similar for both samples, with the largest nanoparticles having a diameter of about 200 nm. However, at higher magnification (Figures 1a and 1c with 5 nm scale), evident differences can be observed. While for the Ag/VC the smallest nanoparticles are in the range of 1 to 3 nm, no nanoparticles of this size were identified in the Ag/MC.

Still, clusters of Ag atoms smaller than 0.5 nm can be found in the Ag/MC sample, and therefore some of the active sites could behave like single-atom-catalysts. Additional HAADF-STEM measurements can be found in Figure S1, highlighting the presence of such Ag atomic clusters. Although X-ray photoelectron spectroscopy (XPS) results do not clearly show evidence of Ag clusters bound to nitrogen centers, their presence cannot be discarded.^38^ These differences in the morphology of the nanoparticles in the lower scale impact the activity and stability of these materials, as will be presented in the following sections.

### 3.2 Cyclic Voltammetry

**Figure 2:**
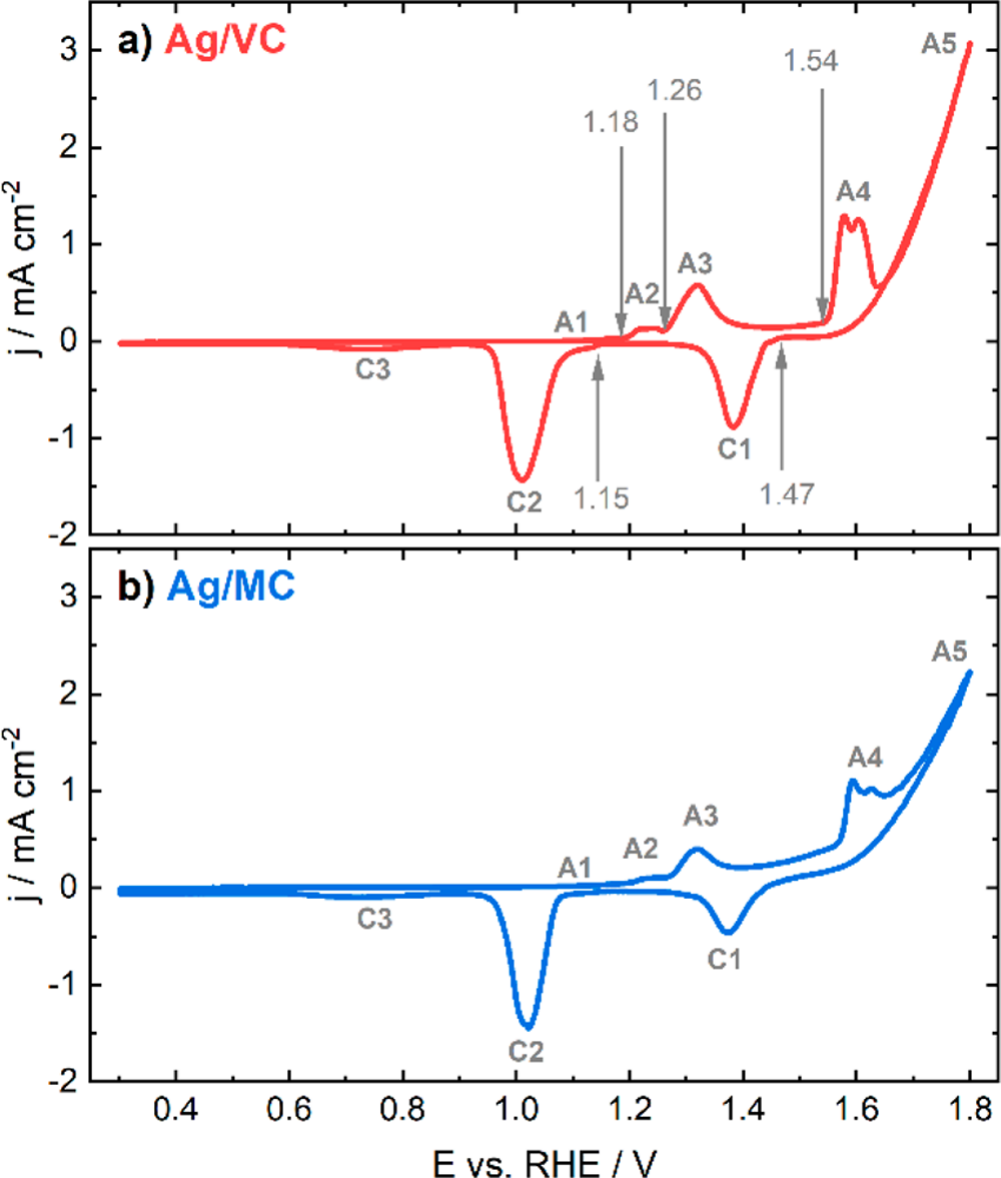
Comparison of the first cyclic voltammogram of Ag/VC (a) and Ag/MC (b) in Ar-saturated 0.05 M KOH, for which the potential was cycled between 0.3 and 1.8 V vs RHE; scan rate: 2 mV s^–1^. The arrows indicate the onset potentials for the most relevant anodic and cathodic peaks, respectively, which can be considered approximately the same for both Ag/VC and Ag/MC (A2, A3, A4, C1, and C2).

First, cyclic voltammetry (CV) profiles were measured for both Ag/VC and Ag/MC materials in Ar-saturated 0.05 M KOH to identify the surface redox processes that will be later correlated with the dissolution phenomena. The concentration of KOH was used for allowing a comparison with the following online ICP-MS dissolution results, since higher concentrations cannot be employed due to technical limitations of the instrument. A potential window between 0.3 and 1.8 V vs RHE was chosen for covering all the range within oxidation and reduction processes of Ag that can take place. These measurements were performed with the RDE configuration for ensuring complete deoxygenation of the solution. The CV curves are displayed in Figure 2.

**Figure 3:**
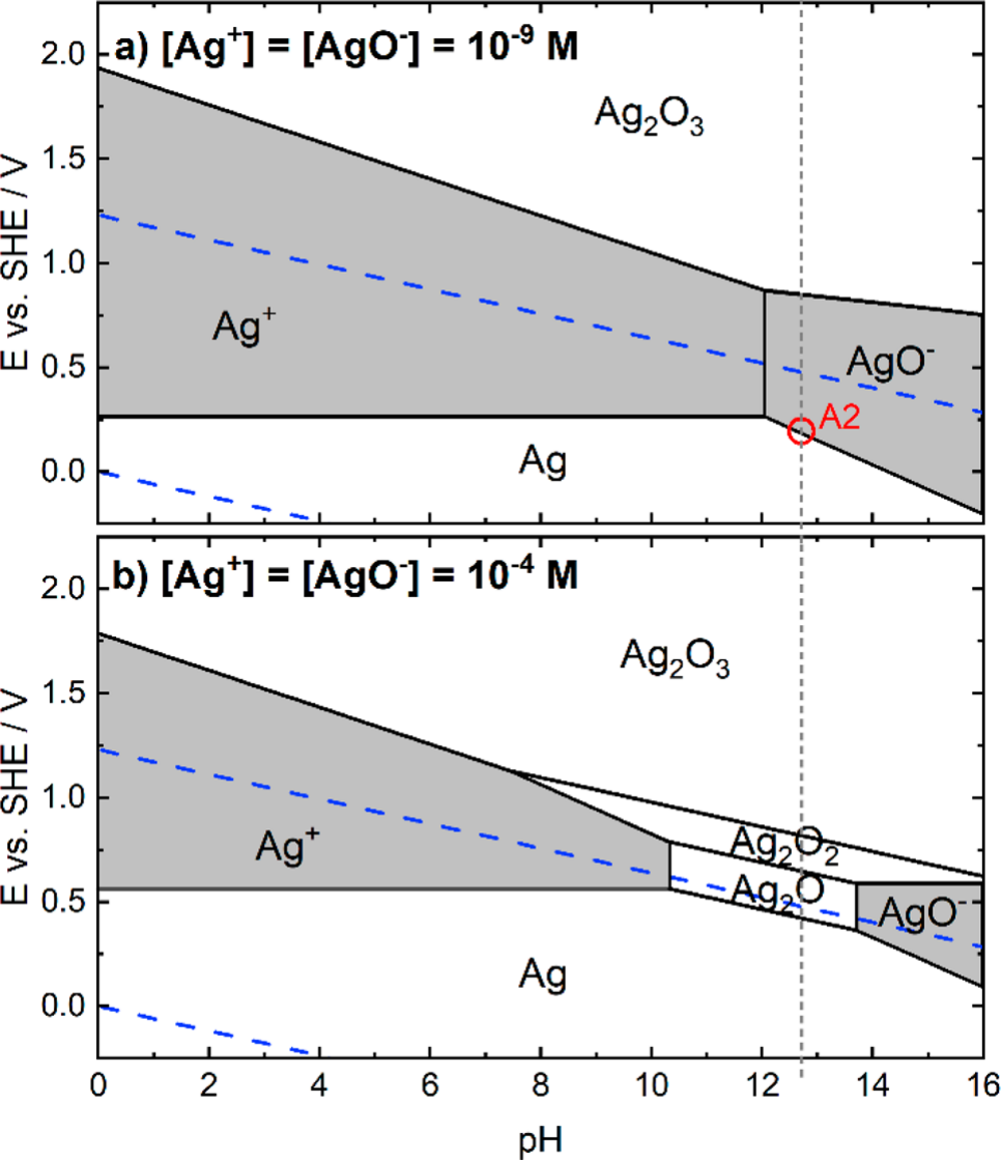
Pourbaix diagram for Ag considering as concentration for the soluble species 10^–9^ M (a) and 10^–4^ M (b). Soluble species are highlighted with a gray background. The vertical dashed gray line marks the working pH in this work (12.7). The red circle in panel a indicates the potential at which the AgO^–^ would be formed, which correlates well with peak A2 in the cyclic voltammetries. The blue dashed lines display the water stability limits.

The observed anodic peaks are labeled from A1 to A5, while the cathodic peaks are named from C1 to C3. The corresponding onset potential values for the most relevant peaks are indicated with arrows. On the one hand, the origin of the peaks A1 and A2, which correspond to the initial stages of silver oxidation, is not totally clear. Peak A1 was ascribed to the dissolution of silver in the form of Ag(OH)_2_^–^ (eq 1) and the formation of a first monolayer of Ag_2_O (eq 2).^29,44,45^ Different origins of peak A2 were proposed: (i) the formation of Ag(OH) (eq 3), which in turn can dissolve in the form of Ag(OH)_2_^–^ or AgO^–^ (eq 4 and 5); (ii) the preferential oxidation of silver atoms with low coordination number; or (iii) the total completion of the Ag_2_O monolayer.^29,46^
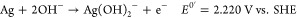
1
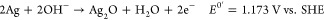
2
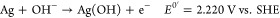
3
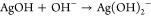
4
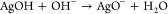
5According to the Pourbaix diagram, the reaction in eq 6 could also be involved in peak A2, although it can be considered just as the sum of eq 3 and 5:
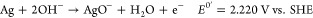
6On the other hand, there is consensus about the reactions that originate the peaks A3, A4 and A5. The peaks A3 and A4 can be ascribed to the formation of bulk Ag_2_O and Ag_2_O_2_, respectively (eq 2 and 7). The peak A5 corresponds to the foot of the OER wave and the formation of Ag_2_O_3_, the highest silver oxide possible (eq 8).^29,47−51^
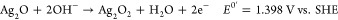
7
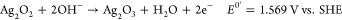
8Figures 3a and 3b show the Pourbaix diagram of Ag considering 10^–9^ and 10^–4^ M as the concentrations for all the possible soluble species. In the first case, the value 10^–9^ M is chosen because it constitutes the estimated lower concentration that can be detected by the ICP-MS.^17^ By using this concentration value, the potential at which the AgO^–^ soluble species starts to form is in agreement with the potential at which the first peaks in cyclic voltammetry appear. In the second case, 10^–4^ is assumed to consider the scenario in which the concentration of the dissolved species near the electrode surface is higher. It can be seen that the order of eqs 2, 6, 7, and 8 is well predicted by the Pourbaix diagram in Figure 3b, although the precise potential values at which they occur may deviate, which could be related to (I) likely different size of particles used in this study and in the reference work^28^ providing thermodynamic data for construction of Pourbaix diagrams (II) kinetic effects.

Regarding the reduction peaks, C1 can be ascribed to the reduction of the highest oxides, Ag_2_O_3_ and Ag_2_O_2_, while peak C2 corresponds to Ag_2_O and Ag(OH) reduction as well as the further reduction of the previous oxides. Finally, peak C3 corresponds to the ORR of the remaining oxygen near the surface from the previous OER in the positive-going scan, since the CVs were recorded in static conditions.^17,29,52^ Correlations between the anodic and cathodic peaks described here with the dissolution processes will be established in the next section with the help of the online electrochemical ICP-MS measurements. The CV profiles corresponding to the exact protocols used for the online dissolution measurements are shown in Figure S2.

### 3.3 Online SFC-ICP-MS

Online dissolution measurements were performed in deoxygenated 0.05 M KOH solution coupling an electrochemical SFC to an ICP-MS for studying the stability of the Ag/VC and Ag/MC materials in a potential range in which surface oxidation–reduction reactions and the OER take place. The time- and potential-resolved obtained signal allows establishing relationships between the dissolution processes and the surface reactions elucidated from the CV profiles in the previous section.

**Figure 4:**
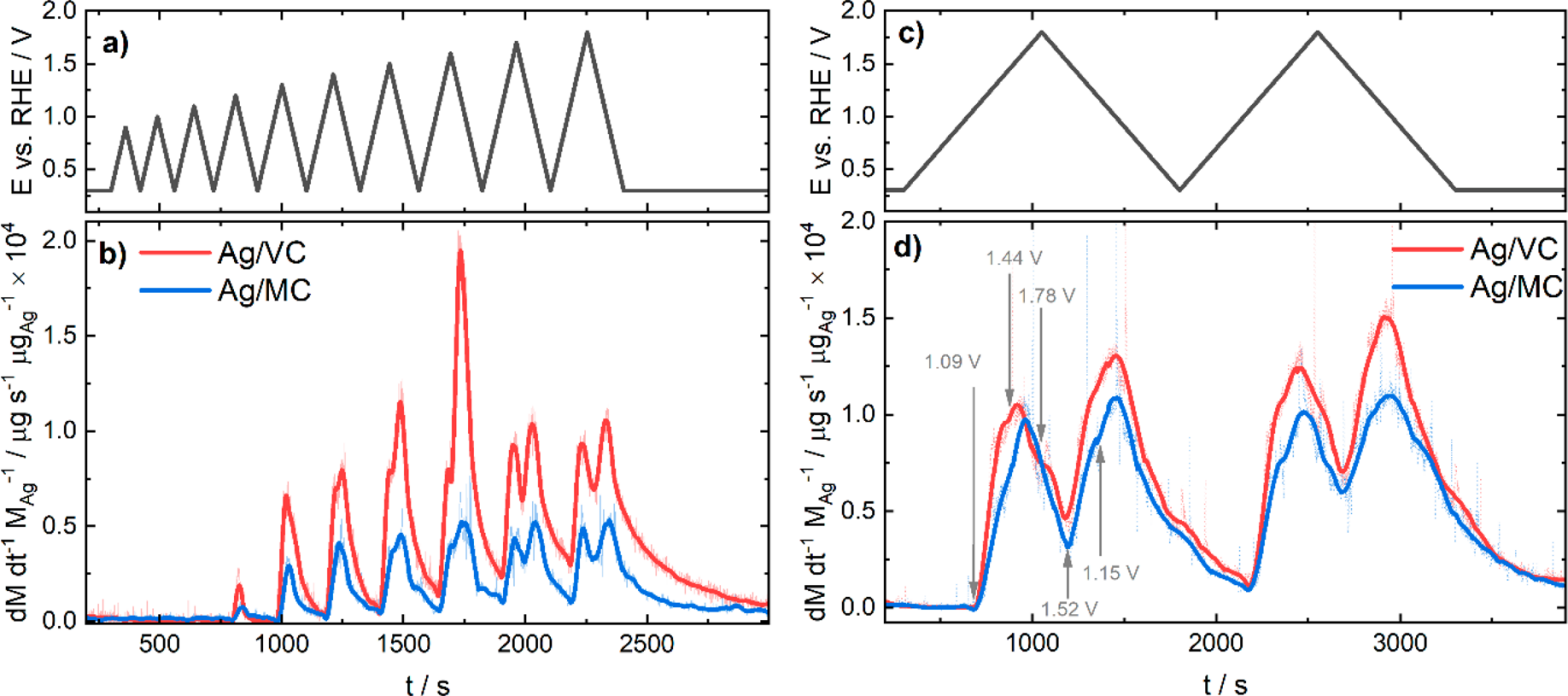
(a) Potential vs time signal for *Protocol 1* and (b) dissolution profiles obtained with the SFC-ICP-MS technique for Ag/VC and Ag/MC catalyst materials in Ar-saturated 0.05 M KOH. (c) Potential vs time signal for *Protocol 2* and (d) dissolution profiles obtained with the SFC-ICP-MS technique for Ag/VC and Ag/MC catalyst materials in Ar-saturated 0.05 M KOH. The arrows approximately denote the onset for the anodic and cathodic dissolution peaks, respectively.

Figures 4a and 4b show the results for electrochemical *Protocol 1*, which consists of consecutive cyclic voltammograms with a scan rate of 10 mV s^–1^ and for which the upper potential limit was increased by 0.1 V after every cycle. The data presented in Figure 4b are normalized to the initial mass of Ag. The non-normalized data can be found in Figure S3. Figure 4a represents the employed electrochemical protocol, while Figure 4b displays the dissolution profiles for Ag/MC and Ag/VC.

The first (anodic) dissolution peak for both catalysts appears for the cycle with *E*_*u*_ = 1.2 V, which would correspond to peak A2 in Figure 2 and therefore to the formation of Ag(OH)_2_^–^ and AgO^–^ dissolved species. For cycles with *E*_*u*_ = 1.4 V or higher, in addition to the anodic dissolution peak, a second (cathodic) dissolution peak can be observed, related to the reduction of the different Ag oxides, first to Ag_2_O and for higher *E*_*u*_ values also to Ag_2_O_2_ and Ag_2_O_3_. This points out that there is cathodic dissolution happening together with the (reduction) reactions (2), (7) and (8), involving the formation of Ag(OH)_2_^–^ and AgO^–^. At the same time, the first anodic peak also increases as *E*_*u*_ is increased, suggesting that anodic dissolution also occurs during the formation of the different Ag oxides. Only two peaks, one for anodic and other for cathodic dissolution, can be clearly distinguished for all *E*_*u*_ values during this protocol, which would comprise together all the anodic and cathodic processes, respectively.

The cathodic dissolution signal keeps increasing up to *E*_*u*_ = 1.6 V, but after this potential, it is reduced, especially for Ag/VC, for which a noticeable diminution in the cathodic dissolution peak is observed. This hindrance to the cathodic dissolution is possibly due to a passivation effect of the newly formed Ag_2_O_3_ layer at about 1.7 V, which would limit the further dissolution of the catalyst. A similar behavior was observed previously for Ir surfaces.^53^ In addition, during this cycle, the anodic and cathodic dissolution peaks are better resolved. For the last cycle with an upper potential limit of 1.8 V the Ag_2_O_3_ layer is fully formed but the dissolution rate remains similar to the previous cycle due to the mentioned passivation effects. The reason for the overshoot in cathodic dissolution for Ag/VC in the case of *E*_*u*_ = 1.6 V and its following noticeable diminution could be due to the different morphology of this catalyst in comparison with Ag/MC, as pointed out by the STEM images in Figure 1. The number of big particles for Ag/VC is higher than for Ag/MC, for which part of the activity contribution comes from Ag atoms clusters. Therefore, the formation of extensive Ag oxides that can give rise to subsequent cathodic dissolution is more favored for Ag/VC, and then the difference in dissolution is more important when the surface of these bigger particles becomes passivated for *E*_*u*_ higher than 1.6 V. In the case of Ag/MC this effect is less noticeable since it contains more very small particles in which the Ag oxides cannot be formed to such a great extent.

A second electrochemical program with a lower scan rate, named *Protocol 2*, was employed to better resolve in time the different dissolution reactions that take place during the potential sweep, and the results are displayed in Figures 4c and 4d. Analogously to *Protocol* 1, the data presented in Figure 4d is normalized to the initial mass of Ag, and the original data is reflected in Figure S4.

In the case of *Protocol 2*, the dissolution rate of Ag is already high during the first scanning cycle between 0.3 and 1.8 V vs RHE for both Ag catalysts. However, Ag/MC shows a slightly higher stability with a Ag dissolution rate of ca. 1.0 μg s^–1^ μg_Ag_^–1^ as compared ca. 1.35 μg s^–1^ μg_Ag_^–1^ for Ag/VC. In addition, the dissolution rate during both cycles with either Ag catalyst stays the same. The onset of dissolution for both materials takes place at ca. 1.09 V, and it is related to peaks A1 and A2 in the CVs (Figure 2), corresponding to the processes described by eq 1 and 6, in which the dissolved species Ag(OH)_2_^–^ and AgO^–^ are formed. The onset potential for the first dissolution peak is also predicted very well by the Pourbaix diagram in Figure 3a.

After this first shoulder, another dissolution peak starts at ca. 1.44 V, coinciding with peak A3 for the formation of Ag_2_O, during which still some anodic dissolution is occurring. This second peak reaches its maximum at ca. 1.65 V, corresponding to peak A4 for Ag_2_O_2_ formation. This means that once the Ag_2_O_2_ layer is formed the surface becomes passivated, and therefore the dissolution rate decreases while going to more positive potentials up to 1.8 V, when the highest oxide Ag_2_O_3_ is formed, which can coexist with the previous oxides and also contributes to the passivation of the surface, although some dissolution occurs while its formed as it can be seen by the apparition of a shoulder in the profile at ca. 1.78 V. During the negative going direction, cathodic dissolution significantly starts at ca. 1.52 V, corresponding to the total reduction of the Ag_2_O_3_ and Ag_2_O_2_ layers (peak C1 in the cyclic voltammetry), during which the release of AgO^–^ species can take place to some extent (eq 9 and 10):
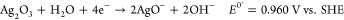
9
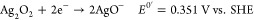
10Finally, a further increase in the dissolution rate is observed at about 1.15 V, which coincides with peak C2, corresponding to the cathodic dissolution from the reduction of the Ag_2_O layer. Cathodic dissolution is maximum at ca. 0.9 V, which is related to the end of the reduction peak C2, and from this value, the dissolution rate goes to zero as the reduction processes of the Ag oxides diminishes their rate to be negligible. It is important to remark that some Ag oxides can still be present on the surface due to their irreversible nature.

All of the onset potentials for anodic and cathodic dissolution are marked with arrows in Figure 4d. The discussion presented here is in agreement with the work by Schalenbach et al.,^17^ although in the latter case they only observed one anodic and one cathodic peaks, which they attributed to eq 6 and 9, respectively. In the present work, a more detailed analysis of the dissolution behavior of Ag is presented which is successfully correlated to the observed peaks in the cyclic voltammograms and the Pourbaix diagram, as discussed in Section 2.1. Therefore, these results contribute to improve the current understanding of the Ag dissolution processes.

**Figure 5:**
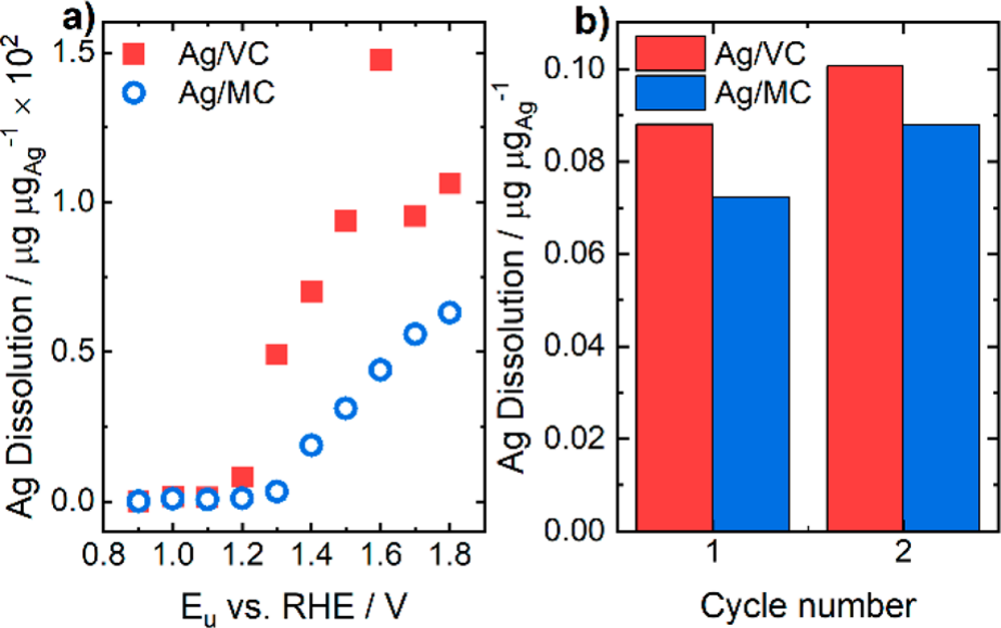
(a) Integrated amounts of dissolved silver vs upper potential limit for *Protocol 1* and (b) integrated amounts of dissolved silver vs cycle number for *Protocol 2*.

Figures 5a and 5b show the amounts of dissolved Ag from the integration of the dissolution rates corresponding to the different upper potential limit for *Protocol 1* and the corresponding cycle for *Protocol 2*, respectively, normalized to the initial mass of Ag. Non-normalized results can be found in Figure S5. When comparing Ag catalysts using *Protocol 1*, Ag from Ag catalyst Ag/MC dissolved 2–3 times less than Ag/VC. Figure 5a clearly shows that after the calculation of the integrated amounts of dissolved silver for each upper potential limit, Ag/VC catalyst, compared to Ag/MC, showed higher dissolved amounts of Ag already at 1.2 V vs RHE. An important decrease in the dissolved amount can be observed from *E*_*u*_ value of 1.6 to 1.7 V due to the passivation effect of the newly formed Ag_2_O_3_ layer, as commented above for Figure 4. From Figure 5 it can be observed that Ag/VC catalyst showed a higher dissolution of Ag compared to Ag/MC. The significantly lower dissolution for the Ag/MC catalyst could be attributed in part to the possible presence of small Ag clusters bound to the N-sites, since nitrogen could provide stability to the metal center as it has been proposed in previous works.^54−57^ Also, the more porous nature of the mesoporous carbon could favor the redeposition of the dissolved Ag^+^ ions. However, to discuss other possible reasons for the higher stability of the Ag/MC material further characterization with IL-TEM will be presented below.

Figures 4 and 5 show that the stability of both catalysts at 1.2 V vs RHE is already compromised, and potential usage in, for example, URFC is questionable due to the instability of the Ag catalyst moving from the ORR potential region toward the OER potential region. Besides the OER potential window, where Ag dramatically dissolves, it is also important to investigate Ag stability in the ORR region of fuel cell mode by using RDE accelerated stress tests (ASTs).

### 3.4 RDE Accelerated Stress Tests

Accelerated stress tests for the working potentials of the ORR up to 1.0 V vs RHE were carried out for both Ag/MC and Ag/VC catalysts for investigating the stability and activity changes during this reaction. A second set of experiments using an upper potential limit of 1.2 V vs RHE was measured to test the stability under harsher conditions, since appreciable Ag dissolution can be observed in Figure 4 at this potential. Incursions into the OER potentials were discarded since dissolution rates are remarkably high at potentials larger than 1.5 V vs RHE, as can be discerned from Figure 4. Additionally, different solution samples were taken during the ASTs to evaluate the dissolved Ag amounts with ICP-MS.

**Figure 6:**
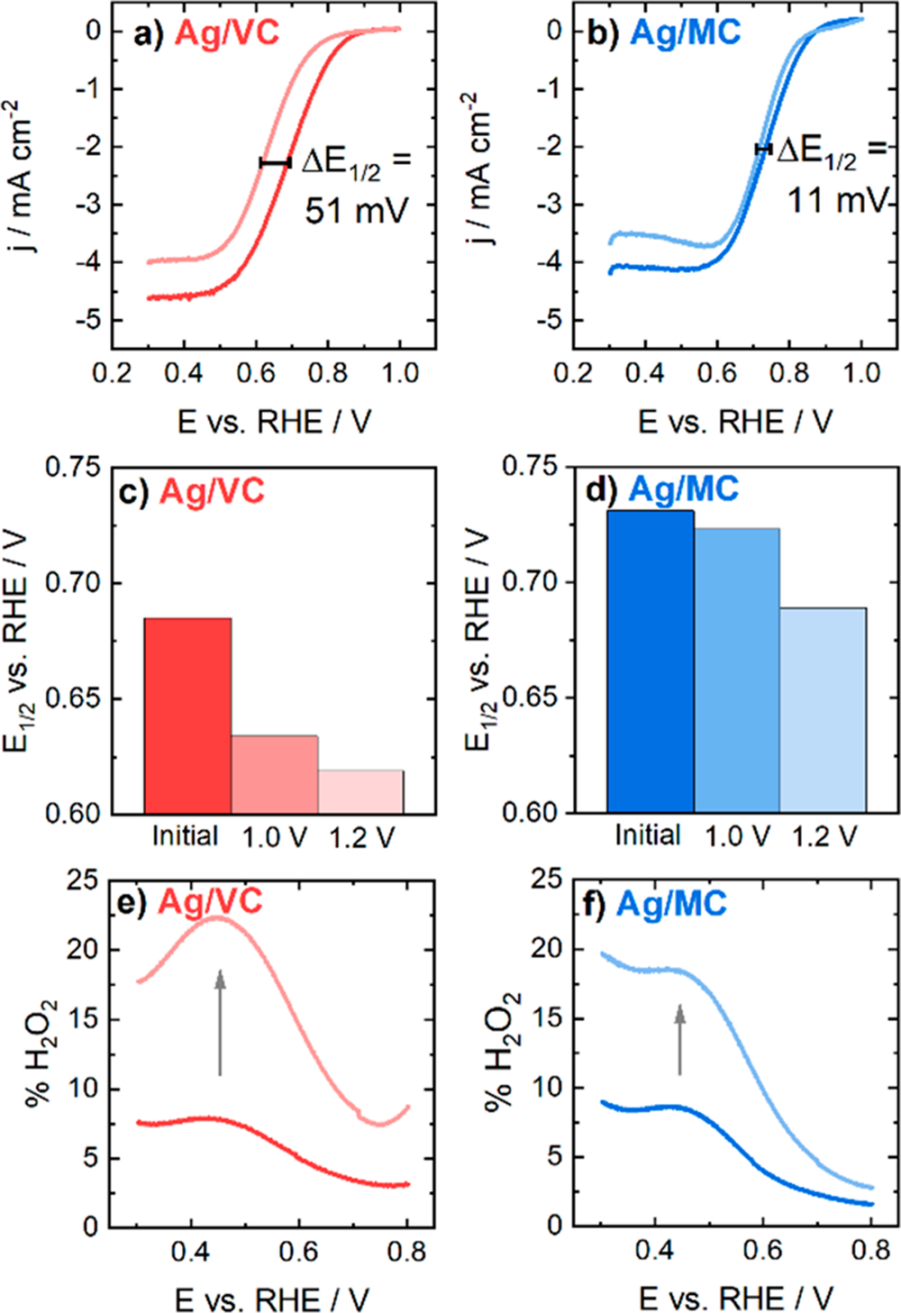
ORR polarization curves for (a) Ag/VC and (b) Ag/MC in O_2_-saturated 0.05 M KOH before and after 10,000 potential cycles (500 mV s^–1^) with upper potential limit of 1.0 V vs RHE, ω = 960 rpm, *v* = 10 mV s^–1^. *E*_1/2_ values for (c) Ag/VC and (d) Ag/MC corresponding to the polarization curves before and after the AST up to 1.0 and 1.2 V vs RHE. H_2_O_2_ yield before and after AST determined from RRDE measurements for (e) Ag/VC and (f) Ag/MC in O_2_-saturated 0.05 M KOH.

Figures 6a and 6b represent the ORR polarization curves within a potential range between 0.3 and 1.0 V vs RHE before and after a complete AST of 10,000 potential cycles in an O_2_-saturated 0.05 M KOH solution using a scan rate of 500 mV s^–1^, and Figure S6 displays the same but for 1.2 V vs RHE as upper potential limit. Figures 6c and 6d depict the half-wave potentials (*E*_1/2_) before the AST, after the AST up to 1.0 V vs RHE, and after the AST up to 1.2 V vs RHE. As shown in Figure 6, for Ag/MC the *E*_1/2_ shifted only by about 11 mV while for Ag/VC the *E*_1/2_ value decreased by about 51 mV. When the upper potential limit was raised to 1.2 V vs RHE the value of *E*_1/2_ shifted negative only by 30 mV for Ag/MC and 60 mV for Ag/VC catalyst. The negative half-wave potential shift for Ag/VC is almost double compared to that of Ag/MC. Therefore, the ORR activity results also indicate that Ag/MC is more stable than Ag/VC, and that Ag/MC is more stable against aggressive conditions.

To check if the observed diminution of the limiting current density is only due to the loss of Ag active surface area or if there is also a change in the H_2_O_2_ yield, the latter was measured by means of RRDE experiments. Figures 6e and 6f point out that there is an increase in the H_2_O_2_ production for both Ag/VC and Ag/MC, which then plays a role in the decrease of the limiting current density observed in Figures 6a and 6b. The H_2_O_2_ percentage yield is similar for Ag/MC and Ag/VC before 10,000 cycle AST test, but the difference in peroxide yield between both materials is more pronounced after the AST. For example, at 0.8 V vs RHE, the H_2_O_2_ percentages after the AST were 8.7% and 2.8% for Ag/VC and Ag/MC, respectively. At 0.45 V vs RHE, both materials show maximum H_2_O_2_ yields of 22.3% for Ag/VC and 18.5% for Ag/MC after the degradation procedure.

The fact that the H_2_O_2_ yield difference is higher for Ag/VC could indicate that the Ag loss in this case is higher, as confirmed by ICP-MS, and therefore, the contribution of the carbon support is higher in this case after the AST, which favors the two-electron transfer process. The Ag/MC method shows lower hydrogen peroxide yield probably because the Ag/MC has a lower Ag loss and an important part of the Ag that remains are clusters bounded to doped nitrogen species which instead promote 4 electron transfer during the ORR process, and VC promotes more 2-electron transfer, thus increasing hydrogen peroxide yield.^38,58,59^ It is important to remark that Ag itself does not always promote the 4-electron pathway and the higher yield of H_2_O_2_ would not be only due to the mass loss of Ag. Carbon supports usually tend to promote 2-electron pathway^60,61^ and thus the formation of higher amounts of H_2_O_2_ when AgNPs fully dissolve. In addition, Ag has different facets that promote either 2 + 2 or direct 4-electron pathways,^62^ and different AgNP sizes have a similar effect.^63,64^

### 3.5 Ag Amounts Dissolved during the Accelerated Stability Test

**Figure 7:**
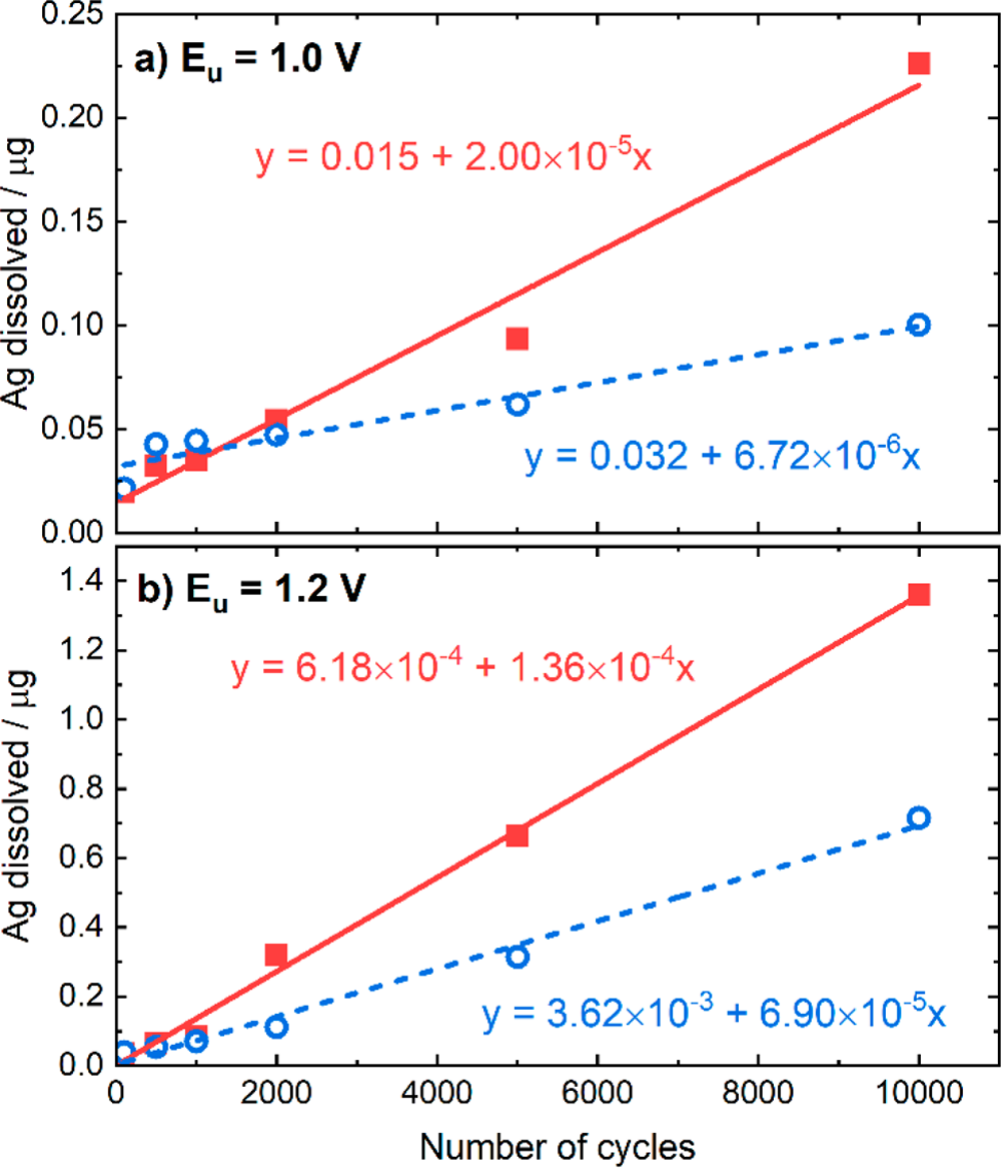
A comparison of Ag dissolved for Ag/VC (red) and Ag/MC (blue) obtained from ICP-MS measurements after ASTs using the RDE technique at different number of potential cycles, using two different upper potential limits: (a) 1.0 V vs RHE and (b) 1.2 V vs RHE. The dashed lines indicate the dissolution trend approximated to a linear regression.

Figures 7a and 7b show the dissolved amounts of Ag after different numbers of potential cycles in an O_2_-saturated 0.05 M KOH solution using a scan rate of 500 mV s^–1^, with upper potential limit of 1.0 and 1.2 V vs RHE, respectively. According to Figure 7a, the amount of Ag dissolved is smaller for the Ag/MC catalyst than for the Ag/VC after 2000 potential cycles. Figure 7b not only suggests similar trends between the two catalyst materials but also points out that raising the upper potential limit by only 0.2 V has a tremendous effect on the amount of Ag dissolved. More precisely, the increase in the upper potential limit has more than quadrupled the dissolution rate and thus the amount of Ag dissolved. Therefore, the stability of Ag catalysts is significantly compromised already when reaching potentials of 1.2 V vs RHE.

For both Ag/MC and Ag/VC catalysts, the difference in Ag dissolution is most visible after the 2000th cycle. In addition, when the upper potential limit was 1.0 V vs RHE the largest difference was at the 10,000th cycle where the difference of the amount of Ag dissolved between the catalysts was doubled. When the upper potential limit was increased to 1.2 V vs RHE, the difference in the Ag dissolution rates more than doubled already at the 2000th cycle.

The dissolved amounts have been approximated to a linear trend, and the corresponding equations are shown in Figure 7. According to these equations and considering that initial loading of Ag in the RDE experiment on the GC electrode is 12 μg, in the case of the 1.0 V protocol it would take 300,000 cycles to dissolve 50% of the catalyst for Ag/VC while in the case of Ag/MC it would take 890,000 cycles. The previous cycles can be considered as shut-off/shut-events in a fuel cell operated with these catalysts.

Considering 3 cycles per day, this value corresponds to 100,000 days and 300,000 days for Ag/VC and Ag/MC, respectively. For the case of the 1.2 V protocol, the ASTs can be compared with cycles with incursions to 1.2 V, which can take place when there is a potential spike during the operation of a fuel cell. In this case, 50% of the catalyst would be dissolved in 44,000 cycles for the Ag/VC and 90,000 cycles for the Ag/MC. Assuming again 3 cycles per day, those number of cycles correspond to 15,000 and 30,000 days, respectively.

It is important to note that these measurements were carried out in 0.05 M KOH solution for allowing subsequent measurements with ICP-MS, but this concentration is not common for real-life devices, and the conditions in a practical fuel cell are more aggressive than the ones in an aqueous model system like the RDE, and therefore, one could expect lower lifetimes in real-life conditions. Just to compare, in a previous work for Pt nanoparticles using the SFC-ICP-MS technique in acid media, it was stated that it would take more than 20,000 cycles to dissolve half of the catalyst when an upper potential limit of 0.95 V vs RHE was used.^65^ Considering that in this work we determined ca. 900,000 cycles for Ag/MC, the stability of this material would be suitable for its use as a cathode material in a fuel cell. Finally, the presented dissolution results remark on the higher stability of the Ag/MC catalyst compared to that of the Ag/VC catalyst.

### 3.6 RDE Identical Location TEM (IL-TEM) Measurements

It was pointed out from both the online and *ex situ* RDE dissolution measurements with ICP-MS that the Ag/MC material is more stable than the Ag/VC. To investigate into possible reasons behind this behavior, RDE-IL-TEM measurements were carried out, since they can provide information about the surface morphology changes of the electrocatalysts during the AST protocols for ORR.

**Figure 8:**
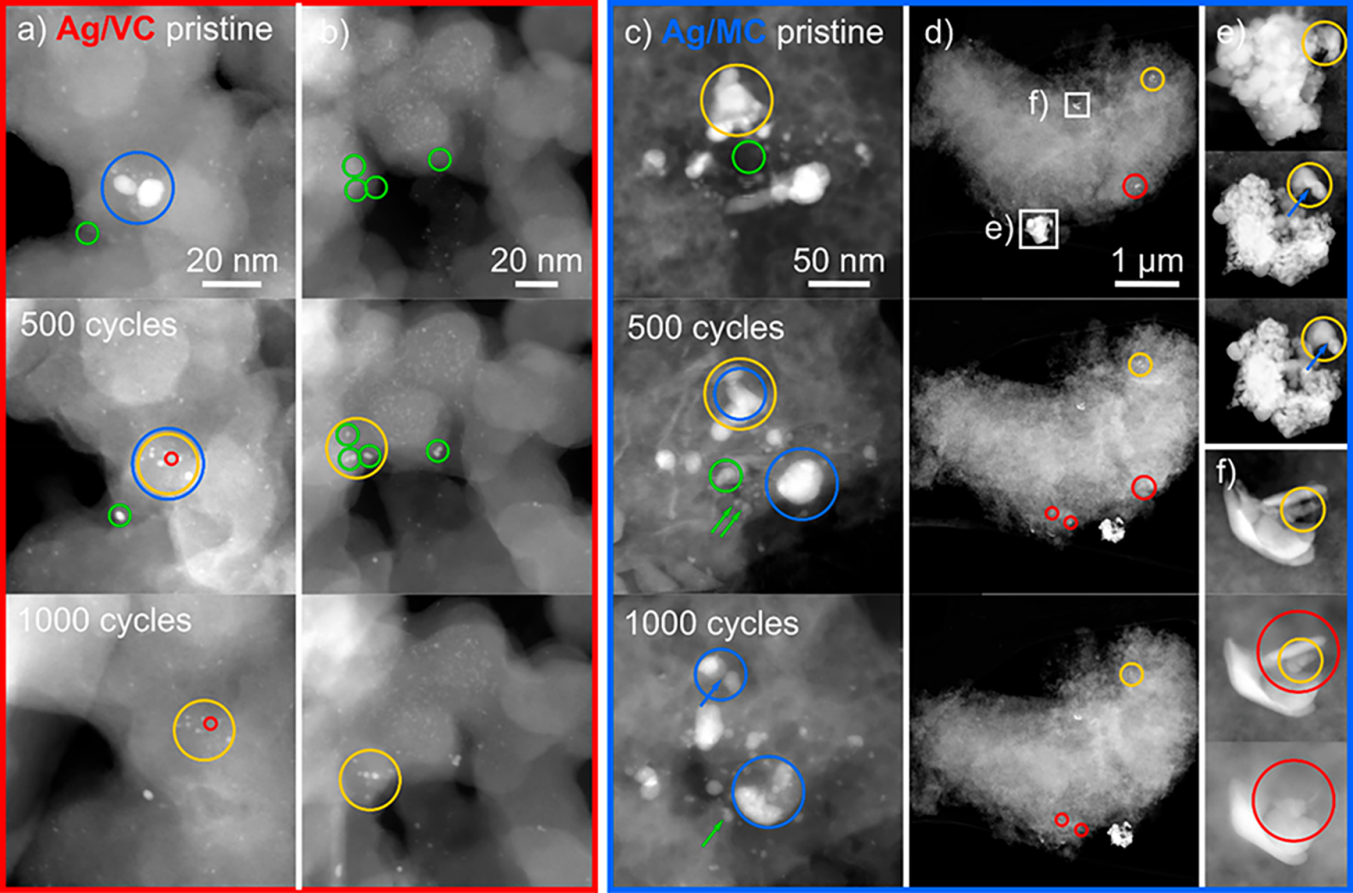
Exemplary RDE-IL-TEM measurements for Ag/VC (a, b) and Ag/MC (c–f) for the pristine sample (top), the sample after 500 cycles of AST (center), and the sample after 1000 cycles of AST (bottom). Green circles: Appearance of particles. Yellow circles: Diminution of size of particles. Blue circles: Splitting of particles into smaller ones. Red circles: Disappearance of particles.

The TEM grid was first covered with a catalyst ink and imaged to find areas of interest (top row in Figure 8). After that, the grid was placed in the RDE holder for electrochemical tests and subjected to the first 500 cycles of AST. Then, the grid was remeasured with IL-TEM to observe changes in the catalyst morphology (center row in Figure 8). The same procedure was carried out after subsequent 500 AST cycles (bottom row in Figure 8).

For the Ag/VC material, some nanoparticles continuously decrease in size (yellow circles). In addition, the green circles point out the appearance of smaller nanoparticles, which probably stem from redeposited Ag that may originate from previously dissolved particles.

Regarding the Ag/MC material, the red circles highlight that nanoparticles disappear during the AST protocol. There are also regions where the nanoparticles reduce their size (yellow circles) or new nanoparticles appear/grow (green circles), analogously to the case of the Ag/VC catalyst. Growth could be caused by Ostwald ripening or redeposition when adjacent particles dissolve (green arrows). The blue circle highlights a region in which the splitting of big nanoparticles into smaller ones occurs in parallel. In the case of the Ag/MC material, attention has been given to the borders of the catalyst particles, since it is difficult to extract clear conclusions from the regions inside the carbon support due to the possibility of having different focus planes.

To underline the representative nature of these observations, additional measurements in other regions are shown in Figure S7. Notably, these trends not only do hold for the particles exemplarily highlighted in Figures 8 and S7 but also do hold when analyzing particle ensemble size distributions. As depicted in Figure S8, the particle size distributions of both material systems undergo a transition toward a multimodal shape, suggesting again a distinct degree of Ostwald ripening. Likewise, the subsequent growth (etching) of large Ag crystals on VC (MC) is pictured by the distribution. Further elucidation of the distributions can be found in the Supporting Information.

The RDE-IL-TEM results clearly show that parts of the dissolved Ag are redeposited in the carbon support in both samples. In the case of Ag/VC, the loss of activity could mainly come from the remarkable Ostwald ripening, which makes losing a high number of smaller particles and therefore having particles with considerably bigger size and a consequent diminution of the active surface area after the AST. The loss of Ag is lower for the Ag/MC catalyst due to the decreased degree of Ostwald ripening and the initially higher number of very small nanoparticles. Dissolution affects the catalyst activity to a lower extent since higher redeposition is also observed in this case. Furthermore, it can be observed that despite the smaller size of the nanoparticles for Ag/MC, a large number of them remain almost unaltered, probably due to the stabilization effect of the nitrogen sites to these small Ag atomic clusters.

## 4 Conclusions

In this work, the mechanism of carbon-supported Ag dissolution was studied by online SFC-ICP-MS measurements, and the results allowed establishing relationships between the voltammetric peaks and the dissolution signal for these electrocatalysts, expanding the current knowledge about Ag stability and its relationship with surface oxidation. The remarkably high dissolution observed for potentials above 1.2 V vs RHE makes unfeasible the applicability of the materials studied in this work for working as an OER catalyst in a URFC. Complementarily, long-term ex situ RDE experiments indicate from both activity and ICP-MS dissolution results that the Ag/MC is 2–3 times more stable than the Ag/VC catalyst.

From a more practical point of view, in the conditions of the RDE aqueous model system, it can be determined that the Ag/MC material would stand 890,000 shut-off/shut-on fuel cell cycles, while this number would be reduced to 90,000 cycles if potential reaches 1.2 V vs RHE. These numbers suggest that the Ag/MC sample could exhibit good stability as cathode material for fuel cells. Additional RRDE experiments demonstrated that the H_2_O_2_ yield increased almost a 5% more for Ag/VC, which agrees with a higher loss of Ag and more exposed carbon support on which the ORR occurs through a 2-electron pathway.

Finally, HAADF-STEM characterizations were carried out to gain insights about the morphology evolution of the catalysts during operation. The initial imaging on the as-prepared samples shows that the smaller Ag nanoparticles for the Ag/VC are in the range from 1 to 3 nm, while in the case of Ag/MC they are Ag atomic clusters smaller than 0.5 nm. Identical-location TEM measurements with RDE suggest that the main degradation mechanism in the case of Ag/VC causes a shrinkage of the biggest particles accompanied by (partial) redeposition of smaller Ag entities. For Ag/MC, the disappearance of Ag clusters can be observed, but also splitting of particles and a high extent of redeposition are observed. This may be related to the higher porosity of the carbon support.

The imaging suggests that a higher amount of Ag would be lost from the dissolution of the larger particles of the Ag/VC material, accounting for its lower stability. It can be remarked that a stabilizing effect of the N-sites of the mesoporous carbon bound to the small Ag clusters is possible, which is also supported by the lower H_2_O_2_ yield in the case of Ag/MC as pointed out by the RRDE results.

In conclusion, the present work constitutes an example on how different electrochemical, analytical, and characterization techniques can be used both ex situ and in situ to obtain valuable information about the activity and stability of electrocatalysts of interest for practical applications, and that it is necessary to design further strategies for developing new Ag-based catalysts with improved stabilities to enable their use for real electrodes for the oxygen evolution reaction. It is important to take into account that usually it is not possible to make direct extrapolations from the behavior for aqueous model systems such us SFC and RDE to the one for practical fuel cell devices.^66^ In order to perform measurements related to dissolution and changes of morphology, some optimization and development of new techniques would be necessary for studying directly the applied two-electrode systems.^67,68^ The half-cell gas diffusion electrode (GDE) approach was developed recently to bridge the gap in the research between aqueous model systems and full devices, and it has been successfully coupled online to ICP-MS for Pt and Fe–N–C based catalysts supported on carbon.^69,70^ Further works applying the GDE technique to Ag-based materials and comparing to the activity for 2-electrode systems, which was already measured for the Ag/MC catalyst with a maximum power density of 243 mW cm^–2^,^38^ are planned for the future. The results presented here already represent a valuable source of knowledge about the stability and morphological changes of these catalyst materials, which is not common in the present literature.

## Supporting Information Available

Additional HAADF-HRSTEM micrographs for Ag/MC, cyclic voltammetry profiles for all the steps in the used electrochemical protocols, non-normalized results for the online dissolution experiments, ORR polarization curves for the experiments with UPL = 1.2 V vs RHE, additional RDE-IL-TEM results, and RDE-IL-TEM-based particle size distribution analysis (PDF)
