# Supplementary material for: Stability of Carbon Supported Silver Electrocatalysts for Alkaline Oxygen Reduction and Evolution Reactions
Source: ACS Appl Energy Mater. 2023 Nov 10;6(22):11497–509. doi: 10.1021/acsaem.3c01717 (PMC10685861; doi:10.1021/acsaem.3c01717)
Supplement: Supplementary file 1 — ae3c01717_si_001.pdf [file ae3c01717_si_001.pdf]

SUPPORTING INFORMATION

Stability of Carbon Supported Silver Electrocatalysts for Alkaline Oxygen Reduction and Evolution Reactions

Jonas Mart Linge[a], Valentín Briega-Martos[b,*], Andreas Hutzler[b], Birk Fritsch[b], Heiki Erikson[a], Kaido Tammeveski[a,*], and Serhiy Cherevko[b,*]

[a]*Institute of Chemistry, University of Tartu, Ravila 14a, 50411 Tartu, Estonia*

[b]*Forschungszentrum Jülich GmbH, Helmholtz Institute Erlangen-Nürnberg for Renewable Energy (IEK-11), Cauerstr. 1, 91058 Erlangen, Germany*

[*]*Corresponding authors:*

Valentín Briega-Martos: v.briega@fz-juelich.de;

Kaido Tammeveski: kaido.tammeveski@ut.ee;

Serhiy Cherevko: s.cherevko@fz-juelich.de

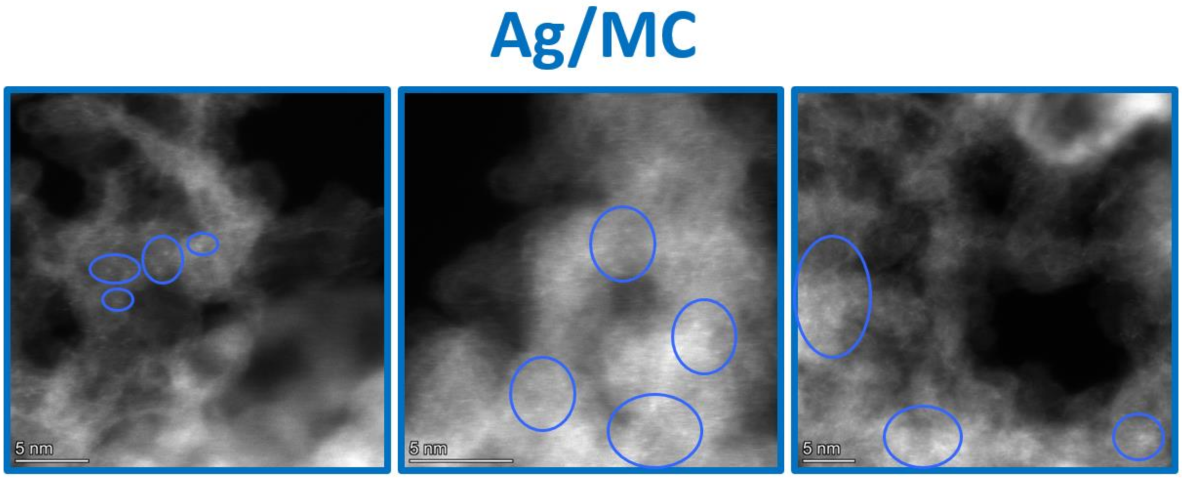

Figure S1. Different HAADF-HRSTEM micrographs for Ag/MC highlighting the presence of Ag atomic clusters smaller than 0.5 nm.

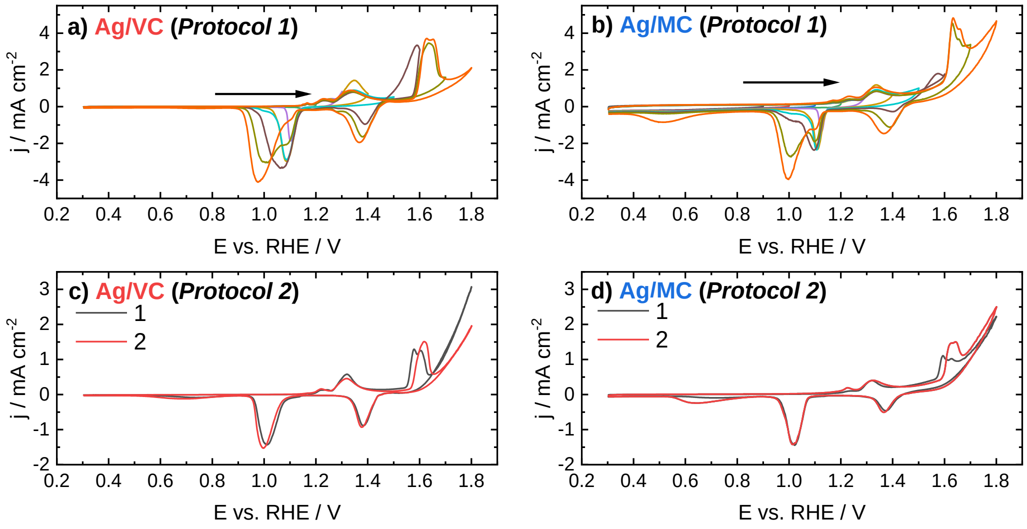

Figure S2. Cyclic voltammetries performed in RDE electrode of Ag/VC and Ag/MC in Ar-saturated 0.05 M KOH, for the exact Protocol 1 and Protocol 2 used for the SFC-ICP-MS measurements.

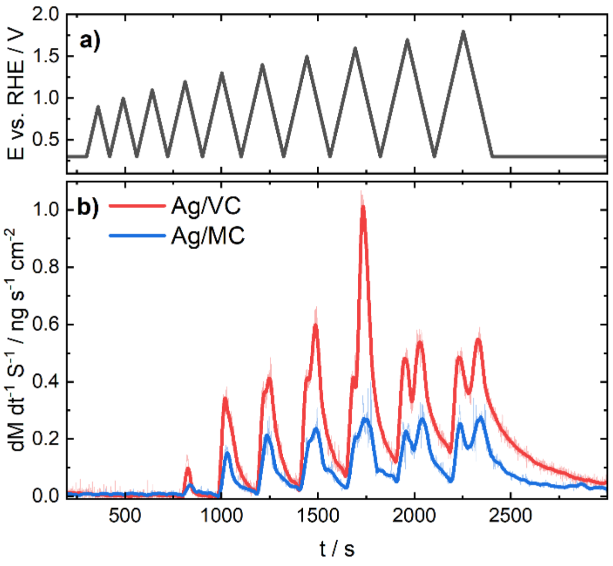

Figure S3. a) Potential vs. time signal for Protocol 1 and b) non-normalized dissolution profiles obtained with the SFC-ICP-MS technique for Ag/VC and Ag/MC catalyst materials in Ar-saturated 0.05 M KOH.

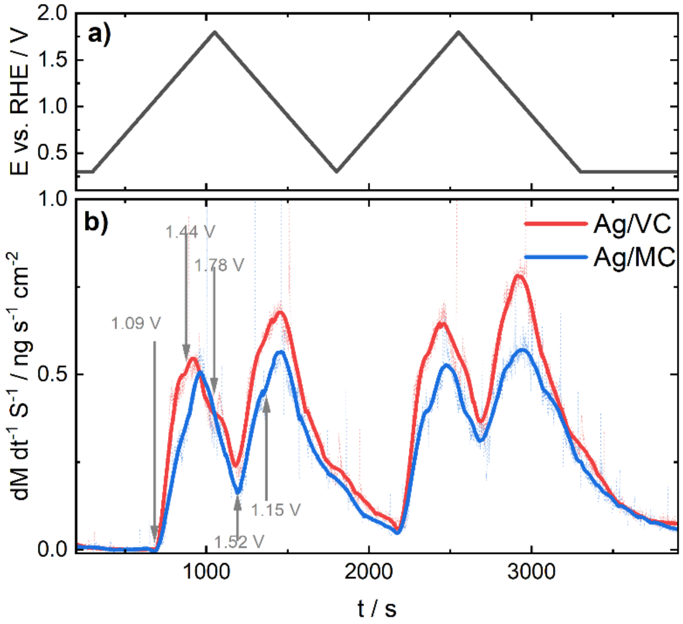

Figure S4. Potential vs time signal for Protocol 2 (top) and non-normalized dissolution profiles obtained with the SFC-ICP-MS technique for Ag/VC and Ag/MC catalyst materials (bottom) in Ar-saturated 0.05 M KOH. The arrows approximately denote the onset for the anodic and cathodic dissolution peaks, respectively.

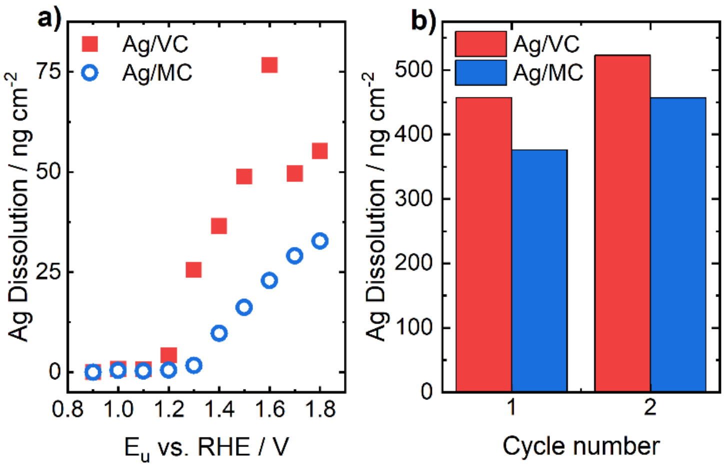

Figure S5. a) Non-normalized integrated amounts of dissolved silver vs. upper potential limit for *Protocol 1* and b) non-normalized integrated amounts of dissolved silver vs. cycle number for *Protocol 2*.

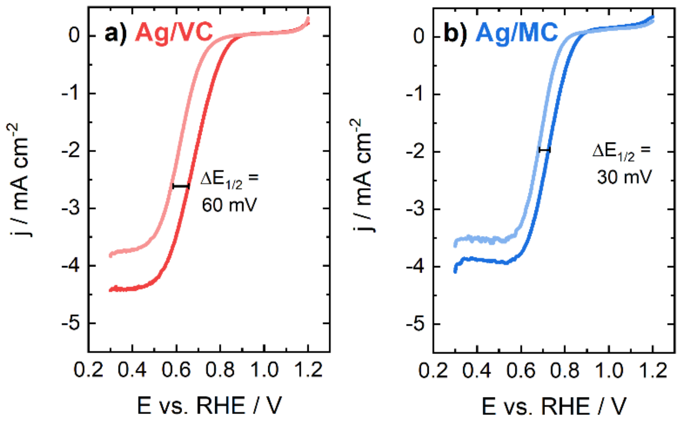

Figure S6. ORR polarization curves for a) Ag/VC and b) Ag/MC in O_2-saturated 0.05 M KOH before and after 10,000 potential cycles (500 mV s^{-1}) with upper potential limit of 1.2 V vs. RHE, ω = 960 rpm, v = 10 mV s^{-1}.

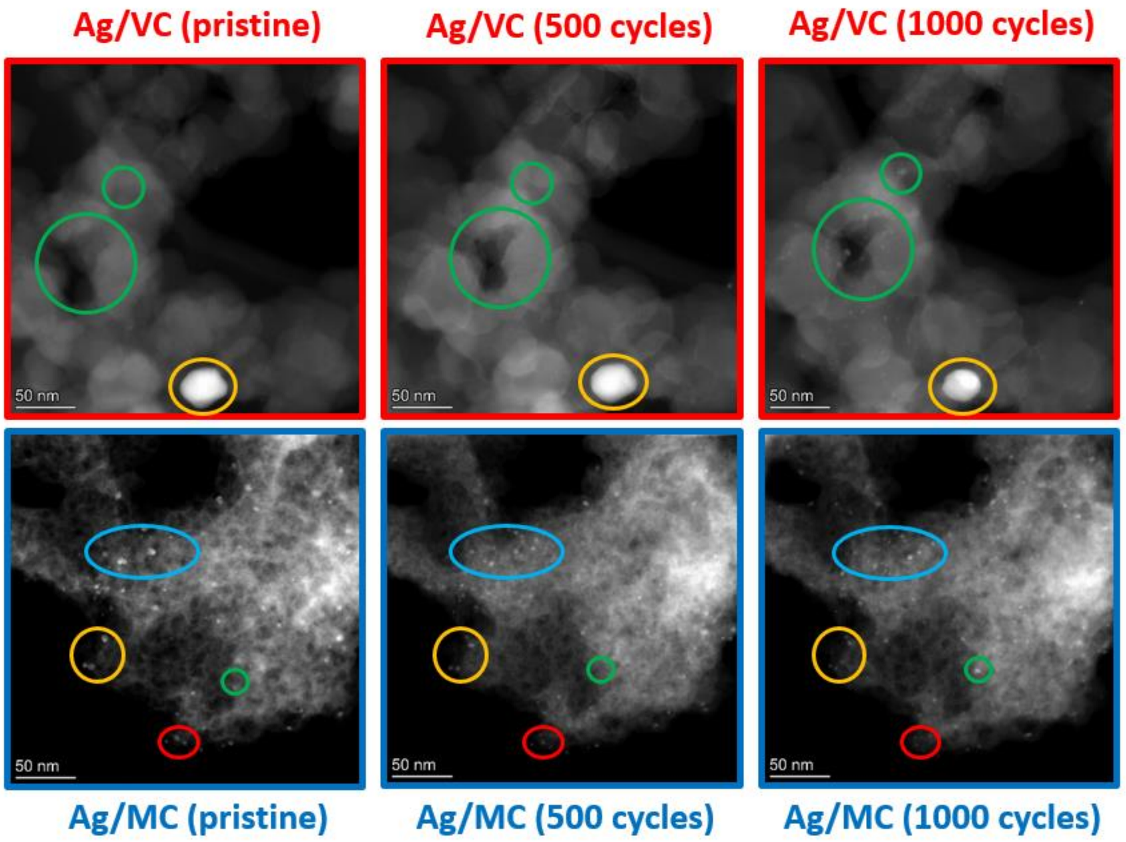

Figure S7. Additional RDE-IL-TEM measurements for Ag/VC (top) and Ag/MC (bottom) for the pristine sample (left), the sample after 500 cycles of AST (center), and after 1000 cycles of AST (right). Green circles: Appearance of particles; Yellow circles: Diminution of size of particles; Blue circles: Splitting of particles into smaller ones; Red circles: Disappearance of particles.

RDE-IL-TEM-based particle size distribution analysis

Particle statistics of IL-TEM measurements were performed using the 'Analyze Particles' function of FIJI.[1] In case of Ag/VC (Fig. S8a), the required binary image was obtained by subsequent median-, Gaussian-, and bandpass filtering, followed by thresholding *via* FIJI's 'intermodes' algorithm on the stack histogram. For Ag/MC (Fig. S8b), the binarization was obtained by subsequent median, and Gaussian filtering, FIJI's 'Substract Backround' algorithm with a rolling ball size of 25 px, and threshold calculation *via* FIJI's implementation of the Otsu algorithm. The latter was performed for every micrograph individually. Only areas with a circularity (as defined by FIJI's Analyzed Particle function) of at least 50% were obtained to mitigate artificial counts.

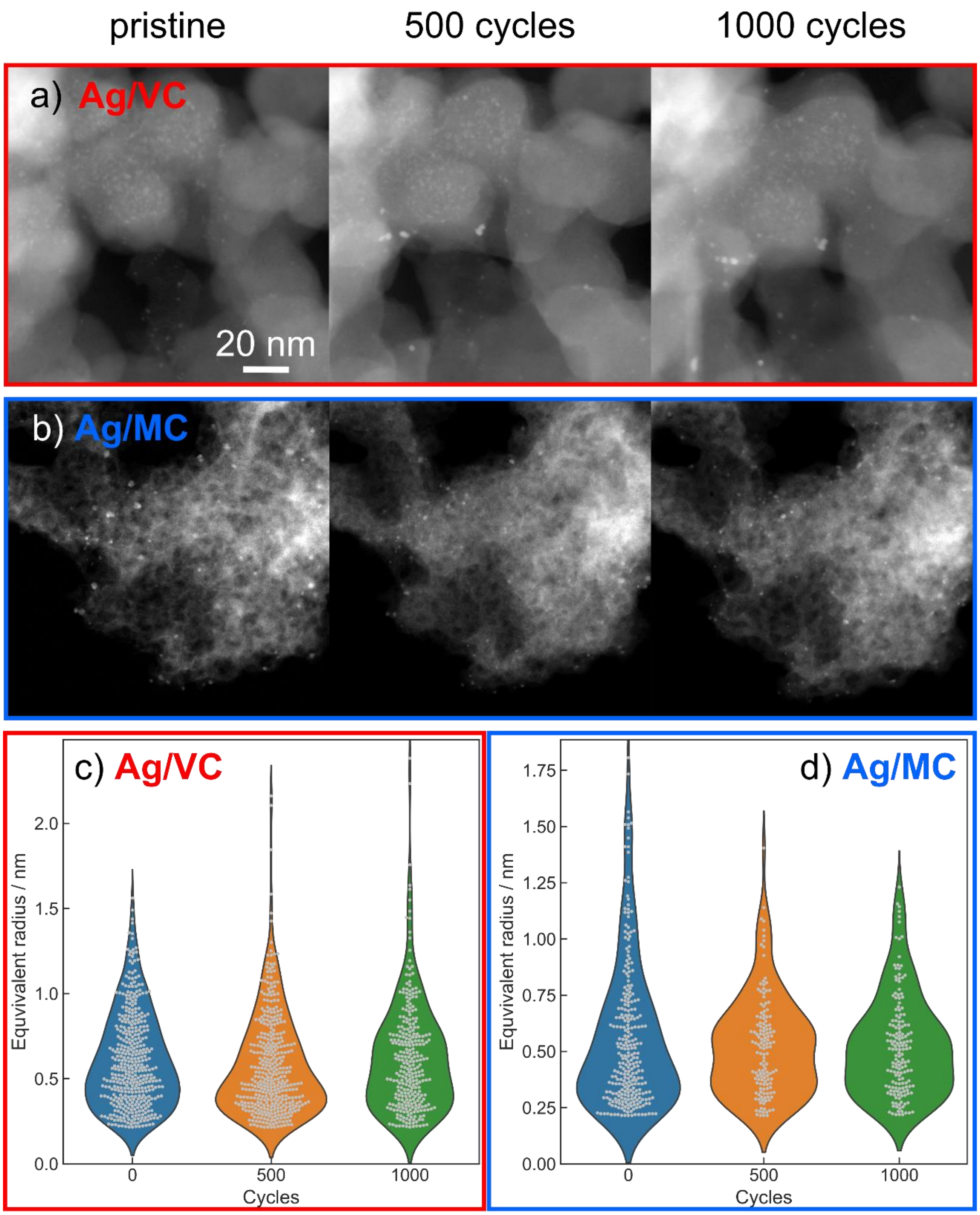

Figure S8: RDE-IL-TEM measurements for a) Ag/VC (reprint from Fig. 9b) and b) Ag/MC (reprint from Fig. S7) alongside with corresponding particle size distributions (c, d).

The obtained particle distributions are displayed in Figure S8c and d, respectively. These violin plots represent kernel density estimation distributions of the particle size evolutions. The particle size is expressed by calculating the equivalent radius of a hypothetical sphere with a similar projected area. The violin plots are overlaid by bee swarm plots where every obtained value is denoted by a gray dot.

Despite a chosen radius threshold of 3 px (about 0.2 nm) and the above-mentioned shape restrictions, small artificial counts cannot be fully excluded *via* this reproducible analysis approach. Thus, information of the particle size distributions close to this lower boundary is limited. Nonetheless, it is visible that for Ag/VC (Fig. S8c) the distribution is shifted towards larger radii, supporting the Ostwald ripening hypothesis stated in the main manuscript. The statistics obtained from the Ag/MC sample exhibits an increased multi-modality of the distribution with cycling which may originate from Ostwald ripening, as well. However, its magnitude appears to be smaller than in the VC case.

References

(1) Schindelin, J.; Arganda-Carreras, I.; Frise, E.; Kaynig, V.; Longair, M.; Pietzsch, T.; Preibisch, S.; Rueden, C.; Saalfeld, S.; Schmid, B.; Tinevez, J. Y.; White, D. J.; Hartenstein, V.; Eliceiri, K.; Tomancak, P.; Cardona, A. Fiji: an open-source platform for biological-image analysis. *Nat. Methods* **2012**, *9* (7), 676-682. https://doi.org/10.1038/nmeth.2019
